# The missing pieces in the catalytic cycle of [FeFe] hydrogenases

**DOI:** 10.1039/d4sc04041d

**Published:** 2024-08-07

**Authors:** Manon T. Lachmann, Zehui Duan, Patricia Rodríguez-Maciá, James A. Birrell

**Affiliations:** a School of Chemistry and Leicester Institute of Structural and Chemical Biology, University of Leicester Leicester LE1 7RH UK prm28@leicester.ac.uk; b University of Oxford, Department of Chemistry, Inorganic Chemistry Laboratory South Parks Road Oxford OX1 3QR UK; c School of Life Sciences, University of Essex Colchester CO4 3SQ UK james.birrell@essex.ac.uk

## Abstract

Hydrogen could provide a suitable means for storing energy from intermittent renewable sources for later use on demand. However, many challenges remain regarding the activity, specificity, stability and sustainability of current hydrogen production and consumption methods. The lack of efficient catalysts based on abundant and sustainable elements lies at the heart of this problem. Nature's solution led to the evolution of hydrogenase enzymes capable of reversible hydrogen conversion at high rates using iron- and nickel-based active sites. Through a detailed understanding of these enzymes, we can learn how to mimic them to engineer a new generation of highly active synthetic catalysts. Incredible progress has been made in our understanding of biological hydrogen activation over the last few years. In particular, detailed studies of the [FeFe] hydrogenase class have provided substantial insight into a sophisticated, optimised, molecular catalyst, the active site H-cluster. In this short perspective, we will summarise recent findings and highlight the missing pieces needed to complete the puzzle.

## Abstract 2

Over the years, spectroscopic, electrochemical and structural studies of [FeFe] hydrogenases have provided ‘pieces’ of information to complete the ‘puzzle’ of their catalytic cycle. However, there are still missing pieces that we need to fill in.

## Graphical abstract

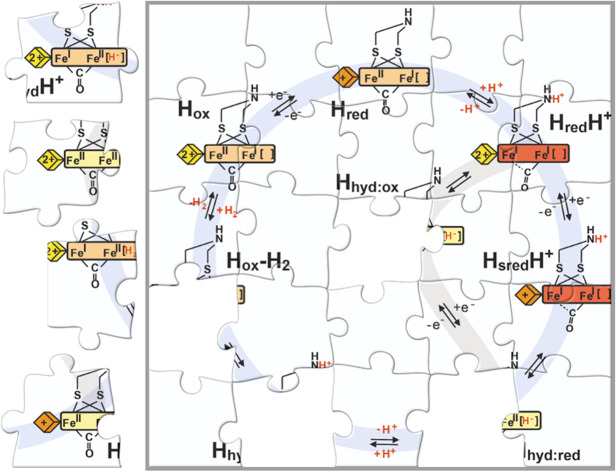

## The big picture: renewable energy and the H_2_ economy

**Fig. 1:**
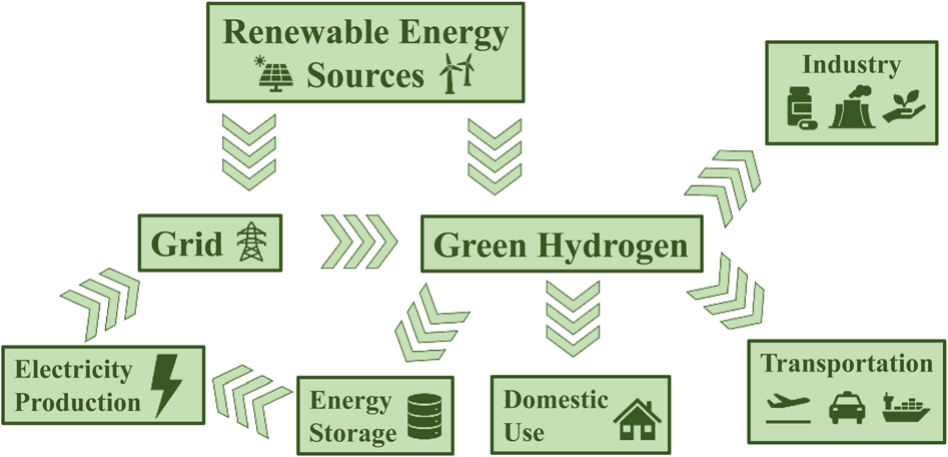
Schematic representation of a hydrogen economy.

The Industrial Revolution marked an incredible time of human ingenuity and progress. Yet, it has also caused huge amounts of carbon dioxide, methane, and other potent greenhouse gases to accumulate in the atmosphere, leading to global warming and climate change. Now, efforts to curb greenhouse gas emissions require us to abandon fossil fuels and move to a completely circular energy economy. While electricity produced by renewable energy sources such as wind and solar power has the potential to supply all our current energy demands, the issue of energy storage remains a difficult problem to solve. Hydrogen could act as a suitable energy vector as it can be produced by water electrolysis and its combustion produces water as the sole byproduct (Fig. 1).^1^ Advantageously, hydrogen is incredibly energy-dense (120 MJ kg^−1^),^2^ however, its storage and transport prove problematic. Regardless, hydrogen is still a crucial reactant for several industrial processes, including oil refining and the Haber Bosch process for the reduction of nitrogen to ammonia, which each use around 30% of annual global hydrogen production.^3^ Presently, 98% of hydrogen is produced from fossil fuels, and of the remaining 2% produced by electrolysis, only a fraction is produced using renewable electricity.^3^

There are currently three major technologies for producing hydrogen *via* electrolysis: alkaline electrolysers, which use nickel-based electrodes and work at very large overpotentials, proton exchange membrane electrolysers, which use platinum, and solid oxide electrolysers, which use nickel-/yttria-stabilised zirconia.^4^ So, while water electrolysis is an established hydrogen production technology, there is still significant interest in developing more efficient, stable, and cheaper relevant materials. Since hydrogen has a relatively large bond dissociation energy (436 kJ mol^−1^),^2^ it is fairly inert and must be activated by a catalyst. As such, there has been incredible investment in understanding how nature uses hydrogen and catalyses this difficult reaction with high efficiency.

In nature, hydrogen is predominantly produced and consumed by hydrogenases, of which there are three phylogenetically distinct classes: the [NiFe] hydrogenases, the [FeFe] hydrogenases and the [Fe] hydrogenases. All three groups heterolytically split hydrogen, with [Fe] hydrogenases requiring a cosubstrate, methenyl-tetrahydromethanopterin, which they hydrogenate by direct hydride transfer. Meanwhile, [NiFe] and [FeFe] hydrogenases reversibly oxidise hydrogen to protons and electrons and transfer these electrons to electron carriers like ferredoxin. Nitrogenases and a plethora of other enzymes have been demonstrated to oxidise or produce hydrogen, albeit at substantially lower rates than the true hydrogenases.^5–11^

Studying how hydrogenases work to understand the crucial mechanistic principles of efficient hydrogen activation has been an active area of research for well over 50 years. But how far have we come and what is left to understand? In this perspective article, we will address these questions, with a particular focus on [FeFe] hydrogenases. We will also discuss where we see the field going over the next years and what the main challenges are for uncovering the ‘missing pieces’ in the catalytic cycle.

## The unique structure of the [FeFe] hydrogenase active site

**Fig. 2:**
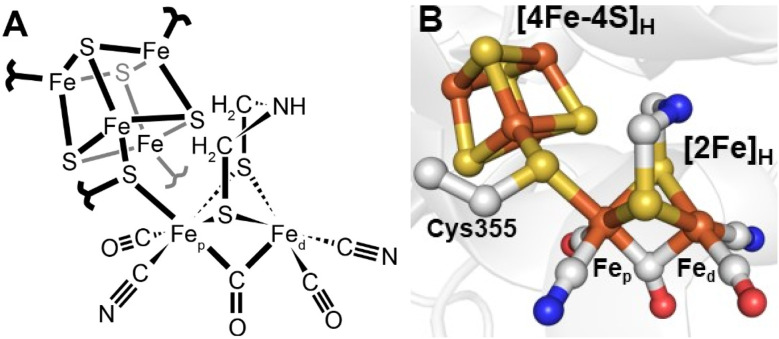
Structure of the active site H-cluster of [FeFe] hydrogenase in (A) a ChemDraw style representation and (B) a Pymol style representation. Residue numbering is from the *C. pasteurianum* enzyme. The image was prepared using PDB ID 4XDC.^24^

Since 1996 it has been known that [FeFe] hydrogenases contain an active site, referred to as the H-cluster, which is a unique type of iron–sulfur cluster coordinated by cyanide and carbon monoxide ligands.^12^ However, its intricacies were unknown^13^ until Peters and coworkers published the X-ray crystal structure of the [FeFe] hydrogenase from *Clostridium pasteurianum* (*Cp*I) in 1998.^14^ For the first time, a unique [2Fe]_H_ subcluster, coordinated by diatomic ligands and an additional unprecedented bridging ligand was observed covalently attached to a [4Fe–4S]_H_ cubane cluster. This was immediately suggested as the site of hydrogen activation. Initially, the unknown bridging ligand was identified to contain two sulfurs, which directly coordinate each Fe. The electron density between them was modelled as water. A later structure from Nicolet and coworkers of the [FeFe] hydrogenase from *Desulfovibrio desulfuricans* (*Dd*HydAB) modelled the bridging ligand as a propane-1,3-dithiolate ligand (PDT).^15^ A subsequent publication from the same group revised this to a 2-azapropane-1,3-ditholate ligand (ADT, also known as di(thiomethyl)amine or DTMA).^16^ This latter idea was supported by some groups through density functional calculations^17^ but others proposed a more likely bridging ligand to be 2-oxapropane-1,3-dithiolate (ODT, also known as di(thiomethyl)ether or DTME).^18^ After years of intense debate, the composition of the dithiolate as ADT is now well established, both through spectroscopic studies^19–22^ and reconstitution studies.^23^ Thus, today we have a fairly clear picture of the active site structure of [FeFe] hydrogenases (Fig. 2).

**Fig. 3:**
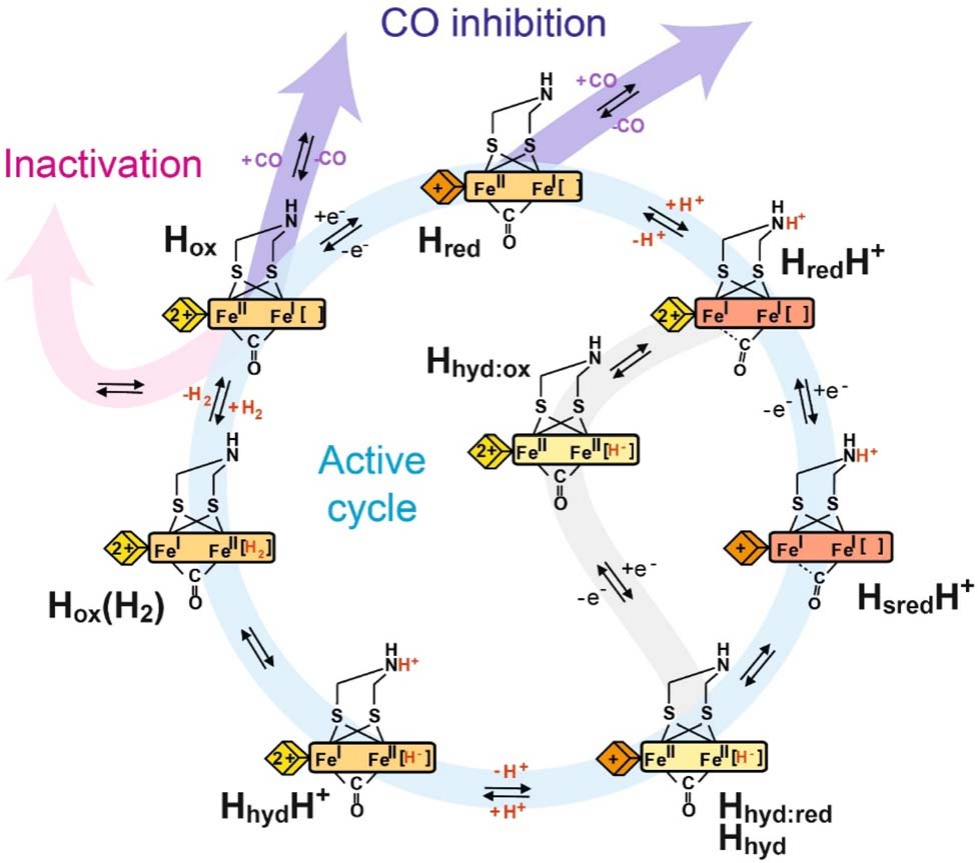
Proposed catalytic cycle of [FeFe] hydrogenase. The blue pathway indicates the active cycle, while the pink pathway indicates inactivation by binding to inhibitors like H_2_S and HCN, the purple pathway indicates reversible inhibition by CO, and the gray pathway indicates an alternative route from the H_red_H^+^ state to the H_hyd:red_ state under less reducing conditions. The yellow, orange and red rectangles represent the [2Fe]_H_ cluster in the Fe(ii)Fe(ii), Fe(ii)Fe(i) and Fe(i)Fe(i) oxidation states, respectively, with the terminal CO and CN- ligands omitted for clarity. The yellow and orange diamonds represent the [4Fe–4S]_H_ cluster in the +2 and +1 oxidation states, respectively.

The [FeFe] hydrogenase H-cluster provides a rare and interesting example of two metal clusters electronically coupled by a simple covalent linkage (Fig. 3). The [4Fe–4S]_H_ cluster in the absence of the [2Fe]_H_ subcluster is stable and functions as a simple redox cofactor.^23^ As is the [2Fe]_H_ subcluster which can also undergo protonation and deprotonation. However, the [2Fe]_H_ subcluster alone is an extremely poor hydrogen conversion catalyst; it is only by inserting it into the [FeFe] hydrogenase protein scaffold that high activity is achieved. Substitution of the sulfur in the [4Fe–4S]_H_ cluster with selenium alters the cluster redox potential but has very little effect on activity.^25^ Meanwhile, mutation of various amino acids surrounding the [2Fe]_H_ subcluster produces highly inactive enzymes,^26^ suggesting this factor is most crucial for enzyme activity. Interestingly though, mutation of the ligands coordinated to [4Fe–4S]_H_ (*e.g.*, from cysteine to histidine) affects activity, shifting the catalytic bias and increasing the overpotential.^27,28^

## General mechanistic principles: proton-coupled electron transfer and hydride formation

In 2006, Roseboom and coworkers investigated the [FeFe] hydrogenase from *Desulfovbrio desulfuricans* using infrared (IR) spectroscopy coupled with electrochemistry.^29^ This enzyme is unusual because it can be purified under air in an inactive over-oxidised state known as H_inact_. In their titrations from high potential to low potential they observed conversion of H_inact_ to an intermediate state H_trans_ followed by formation of the active oxidised state, H_ox_. Further reduction formed the one-electron-reduced H_red_ state, followed by the formation of a two-electron-reduced, or super-reduced, H_sred_ state. Based on the positions of the IR bands as well as the known electron paramagnetic resonance (EPR) properties of each state, the H_inact_ state was described as a [4Fe–4S]^2+^-[Fe(ii)Fe(ii)] state. Reduction to H_trans_ involved reduction of the [4Fe–4S]_H_ cluster and yielded a [4Fe–4S]^+^-[Fe(ii)Fe(ii)] state. H_ox_ was thought to be isoelectronic with H_trans_ with a [4Fe–4S]^2+^-[Fe(ii)Fe(i)] state, where the electron moved from the [4Fe–4S]_H_ cluster to the [2Fe]_H_ cluster. Reduction of H_ox_ to H_red_ was thought to occur at the [2Fe]_H_ cluster yielding a [4Fe–4S]^2+^-[Fe(i)Fe(i)] state, and formation of H_sred_ was thought to involve reduction of the [4Fe–4S]_H_ cluster giving a [4Fe–4S]^+^-[Fe(i)Fe(i)] state. The last transition was observed to be not completely reversible. These transitions were all pH-dependent, indicating the important role of proton-coupled electron transfer for the enzymes' high catalytic activities.

In 2009, Silakov and coworkers performed similar experiments with the smaller, simpler [FeFe] hydrogenase from *Chlamydomonas reinhardtii* (*Cr*HydA1), made similar observations and came to similar conclusions.^30^ However, one interesting finding was a large difference in the IR bands of the one-electron-reduced H_red_ state between the two enzymes. This issue was later resolved when it was discovered that the one-electron reduced state in *Cr*HydA1 consisted of two different states, whose population depended on the pH, having roughly equal populations of both at neutral pH.^31^ Thus, these two states were dubbed the H_red_ and H_red_H^+^ states, where the former was observed predominantly at high pH and the latter at low pH. The H_red_ state had small red-shifts in the IR bands relative to the H_ox_ state whereas the H_red_H^+^ state had large red-shifts, indicating that the H_red_ state had a [4Fe–4S]^+^-[Fe(ii)Fe(i)] electronic structure while the H_red_H^+^ state had a [4Fe–4S]^2+^-[Fe(i)Fe(i)] electronic structure. It was assumed that the ADT ligand bridging the two Fe atoms was protonated in the H_red_H^+^ state facilitating electron transfer by charge neutralisation effects. Using a non-protonatable propane-1,3-dithiolate (PDT) ligand prevented the formation of H_red_H^+^, supporting the assignment.^32^ Furthermore, photolysis experiments were able to enrich the H_red_ state.^33^ Resonance Raman (RR) spectroscopy of *Cr*HydA1 revealed a species containing a reduced [4Fe–4S]^+^ cluster and a H_ox_-like [2Fe]_H_ site, likely derived from H_red_H^+^. The RR experimental conditions, laser illumination at low temperature, could facilitate electron transfer from the [2Fe]_H_ subcluster to [4Fe–4S]_H_, generating a [4Fe–4S]^+^-[Fe(ii)Fe(i)] state, which was named H_red_’ at the time. Katz *et al.* proposed a proton transfer between the H-cluster and a near base (Cys169)^33^ as the driving force for electron transfer between the [4Fe–4S]_H_ and [2Fe]_H_ sites. Therefore, the populations of the isoelectronic states H_red_ and H_red_H^+^ were controlled by the protonation states of the ADT ligand through charge compensation of the [FeFe] subcluster. This was later supported by IR spectroelectrochemical titrations.^31^

Interestingly, the [FeFe] hydrogenase from *D. desulfuricans* only shows the H_red_H^+^ state at neutral pH.^29^ More carefully analysed redox titrations revealed that initial reduction of the H_ox_ state generates a mixture of both H_red_ states but that further reduction converts the H_red_ state into the H_red_H^+^ state. In this case, what appears to happen is the reduction of the accessory iron–sulfur clusters triggers the electron to move from the [4Fe–4S]_H_ subcluster to the [2Fe]_H_ subcluster by charge repulsion. This then increases the pK_a_ on the H-cluster forcing it to be completely protonated at neutral pH.^34^

Despite having very little catalytic activity the [FeFe] hydrogenases in which the ADT cofactor is substituted with PDT or oxapropane-1,3-dithiolate (ODT) have proved incredibly useful for understanding the catalytic mechanism. The PDT variant can only exist in two oxidation states: an H_ox_-like state with [4Fe–4S]^2+^-[Fe(ii)Fe(i)] and an H_red_-like state with [4Fe–4S]^+^-[Fe(ii)Fe(i)]. The missing nitrogen bridgehead seems to prevent the formation of states in which [2Fe]_H_ is reduced to Fe(i)Fe(i) as its reduction potential is too negative without coupled protonation due to charge imbalance. If, as has been suggested by others,^35^ the H_red_H^+^ and H_sred_H^+^ states contain bridging hydrides rather than protonated amines, then it is not clear why these states would not form in the PDT variant; there may be slower kinetics involved but eventually, these states should form. It is also interesting to note that terminal hydrides have yet to be detected in the PDT variant, which hints that the differences between the ADT and PDT variants are more complicated than simply the loss of the bridging amine. It should also be noted that the redox potential for the H_ox_/H_red_ transition has been reported to be pH-dependent, indicating that reduction of [4Fe–4S]_H_ involves PCET.^36^ However, this result has been disputed^37^ and argued to be an artefact due to the presence of sodium dithionite in the preparations.^38^

While the PDT variant was useful for understanding PCET at the H-cluster, the ODT variant has been useful for understanding the formation of terminal Fe-hydrides. The ODT variant forms a fairly stable state with the lowest CO-band at 1867 cm^−1^,^23^ which was thought to have a [4Fe–4S]^+^-[Fe(ii)Fe(ii)]H^−^ structure. The reason for this is still not entirely clear. It has been argued that the ether group in ODT, being much less basic than the amine group of ADT, is a poor proton relay so allows kinetic trapping of the Fe-hydride intermediate.^39^ Another possibility is that, like the PDT variant, the ODT variant does not form the H_red_H^+^ state, but, unlike the PDT variant, the ODT variant undergoes PCET with protonation of Fe_d_. Regardless, Reijerse *et al.* directly observed the terminal hydride on Fe_d_ by studying the ^57^Fe labeled *Cr*HydA1 ODT variant with nuclear resonance vibrational spectroscopy (NRVS).^40^

A final point that required investigation was the observation that both the H_red_H^+^ and H_sred_H^+^ states appeared to be lacking a bridging CO band in the IR spectra, and instead appeared to have an additional terminal CO at approximately 1960 cm^−1^ or 1950 cm^−1^. This finding has been interpreted to mean that the bridging CO becomes terminal leading to the formation of bridging hydride states. Under some circumstances, however, the bridging CO appeared to be retained *e.g.* at low temperature^41–43^ and in sensory enzymes.^44^ Furthermore, the 1960 cm^−1^ and 1950 cm^−1^ bands have been suggested to be due to small amounts of terminal hydride-containing states, which can be populated at low temperatures by photoexcitation. Therefore, the structural assignments of the H_red_H^+^ and H_sred_H^+^ states remain contentious.

Overall, catalysis at the H-cluster appears to proceed as follows: in the proton reduction direction, the active oxidised H_ox_ state can be reduced by one electron. This gives an H-cluster with a pK_a_ of approximately 7.2, so, around pH 7, a protonated (H_red_H^+^) and deprotonated (H_red_) form are observed. The H_red_H^+^ state has an oxidised [4Fe–4S]_H_ cluster and accepts an electron to form the H_sred_H^+^ state. Meanwhile, in the reverse direction, the H_red_ state can be oxidised to the H_ox_ state by electron transfer from the [4Fe–4S]_H_ cluster. This ensures a high level of catalytic reversibility as both redox events happen at very similar potentials, which are pH-dependent and close to the 2H^+^/H_2_ couple. In the H_sred_H^+^ state, both electrons needed for hydrogen production are loaded at the H-cluster and a proton is bound to the [2Fe]_H_ subcluster. Electron and proton rearrangement occurs and the [2Fe]_H_ subcluster is transformed to an isomeric state containing a hydride – the H_hyd_ state. The intermediates of this process were studied by light-induced spectroscopy under cryogenic temperature^43^ (detailed discussion in Section 7). The H_hyd_ state then accepts a second proton forming the transient H_hyd_H^+^ state before hydrogen is formed and released from the active site. After H_2_ is released from H_ox_H_2_, the enzyme returns to H_ox_ and the next turnover begins. This cycle operates in the H_2_ oxidation direction by an exact reversal of each of these steps. In our interpretation of the data, proton-couple electron transfer (PCET) is essential for the catalytic cycle. The [4Fe–4S]_H_ cluster serves as an electron input module and protonation of the amine in the [2Fe]_H_ cluster triggers electron transfer from [4Fe–4S]_H_ to [2Fe]_H_.

## Thermodynamic and kinetic considerations of the catalytic mechanism: pre-steady state and steady-state conditions

So far, most data used to understand the mechanism of [FeFe] hydrogenases have been collected under equilibrium (often near-equilibrium) or steady-state turnover conditions. More *operando* experiments are needed to confirm that the catalytic states identified are indeed catalytic. It has been argued that all states observed under equilibrium/steady-state conditions are thermodynamic sinks and, therefore, off-pathway non-catalytic intermediates.^35,45,46^ This logic would seem to contend that an enzyme with only catalytic states and no off-pathways would be spectroscopically silent, which is extremely unlikely. The energy landscape during catalysis is unlikely to be entirely flat and homogenous, thus, the most abundantly observed intermediates will be those that fall into energy troughs. It should be noted that under equilibrium conditions, a relatively small difference in Gibbs free energy (≈2.7 kcal mol^−1^ – on the order of a hydrogen bond) is required for a state to be 100-fold more abundant than another (eqn (1)).1
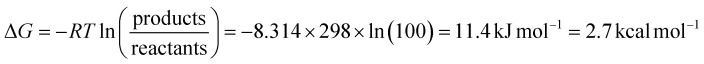


Small energy differences may be responsible for some catalytic intermediates remaining unobserved. However, that does not mean that every observed state is non-catalytic *a priori*. Steady-state turnover may reveal further catalytic states as rate-limiting steps start to populate higher energy intermediates, which accumulate faster than they decay. However, the ultimate proof of a state being catalytic will be its observation during pre-steady state kinetic analysis; the states that appear and disappear within a single turnover must, by definition, be involved in the catalytic cycle.

Despite the importance of such studies, relatively few publications have attempted to measure pre-steady state kinetics due to their experimental challenges. The [FeFe] hydrogenase from *D. desulfuricans* is suggested to turnover at least 10 000 times per second.^47^ This indicates a catalytic cycle spanning 100 μs and requires a time resolution of at least 1 μs. This can be achieved by some spectroscopic techniques, including pump-probe IR spectroscopy to study the CO and CN band vibrations.^48^

On top of this, experiments must be performed under conditions that allow turnover. This precludes (to a certain extent) the use of many spectroscopies (*e.g.* EPR spectroscopy) that require frozen samples. Turnover cannot be limited by exogenous factors such as diffusion of substrates to and from the active site. This adds complication as, during proton reduction, both protons and electrons are required by the hydrogenase. The latter requires an electron mediator, while the former can be mediated by water and buffer salts. Typical diffusion coefficients of small molecules in aqueous solution are on the order of 10^−10^ to 10^−9^ m^2^ s^−1^, while for protons they are estimated to be 10^5^-fold higher based on the Grotthuss proton-hopping mechanism.^49^ Therefore, for efficient (sub-μs) electron transfer *via* an electron mediator, the average distance between the reduced mediator and the enzyme needs to be extremely short and the concentration of both the enzyme and electron mediator must be very high.

Several studies have attempted to measure pre-steady state kinetics in [FeFe] hydrogenases using photosensitisers and diffusible electron mediators for intermolecular electron transfer.^48,50–55^ However, diffusion limitations may still be an issue. To overcome these limitations, the hydrogenase needs to be covalently attached to the photosensitiser so that rapid intra-molecular electron transfer occurs. Several early studies indicated that this would be possible using thiol linkers^56,57^ but so far time-resolved studies using this approach have not been published. An alternative approach is to use the photosensitivity of the H_ox_–CO state. [FeFe] hydrogenases are inhibited by CO, forming the H_ox_–CO state, which is also reducible to the H_red_–CO state. The H_ox_–CO state is known to be photosensitive (at least in the frozen state). An [FeFe] hydrogenase sample prepared under CO in the presence of H_2_ (to initiate H_2_ oxidation) or another reductant (to initiate H_2_ production) might be expected to become catalytically active upon photolysis of the Fe–CO bond. An open question here is whether CO rebinding is more rapid than catalysis.

## Primary coordination sphere and contributions from the protein framework: from secondary coordination sphere to long-range interactions, proton transfer pathway and electron relay

Metallic Fe as well as various synthetic Fe-based materials do not come close to the efficiency or reversibility that is achieved by [FeFe] hydrogenases for hydrogen interconversion. The primary coordination sphere of the H-cluster finely tunes the electronic structure of the Fe ions in such a way that low oxidation states and spin states are stabilised in the enzyme. This is achieved through the combination of strong σ-donating (CN^−^) and π-accepting (CO) ligands, along with the covalent “soft” thiolate ligands. The π back-bonding interactions favor the lower valence Fe(i) and Fe(ii) formal oxidation states as electron density is accepted from the metal 3d_*xy*, *yz*, *xz*_ orbitals. Meanwhile the CN^−^ ligands, which only weakly engage in π backbonding, but are much stronger σ donors, donate electron density into the metal 3d_*x*^2^−*y*^2^_ and 3d_*z*^2^_ orbitals, favoring higher valence formal oxidation states. It was recently observed that the binding of CN^−^ to the H-cluster stabilised its overoxidised Fe(ii)Fe(ii) oxidation state,^58,59^ whereas CO binding maintains the Fe(ii)Fe(i) oxidation state observed for H_ox_. It has not yet been possible to synthesise a [2Fe] analogue with extra CN^−^ ligands, however, it has been possible to integrate a [2Fe] analogue where a CN^−^ ligand was replaced by a CO ligand (*i.e.* MonoCN).^60,61^ In this case, spectroscopic data indicated stabilisation of the Fe(i)Fe(i) oxidation state. These results support the postulation that the balance of CN^−^ and CO ligands in the H-cluster dramatically affects its electronic structure. There is a balance to be struck between the σ-donating and π-accepting character of the ligands to tune the electron density on the metals such that the redox transitions (II/I) fall into a potential range suited for hydrogen production and oxidation.

Additional insight into this can be garnered from studies of synthetic complexes integrated into [FeFe] hydrogenases. In 2015, Siebel *et al.* attempted to reconstitute the H-cluster using 15 synthetic [2Fe] analogues, including the native precursor complex, as well as variations on the dithiolate bridging ligand and the number of CO/CN^−^ ligands.^61^ Ten of these complexes were successfully inserted. Only two of the ten reconstituted enzymes showed significant catalytic activity, namely, the native ADT cofactor and a monocyanide (pentacarbonyl) form of the ADT complex, which presented with 47% H_2_ production activity and 41% H_2_ oxidation activity compared to the native enzyme (Table 1). Evidence provided by Lorenzi *et al.* suggests that the CN^−^ ligand coordinates to Fe_p_ in the monocyanide variant of *Cr*HydA1.^60^ So, although changing the relative σ-donating/π-accepting abilities of the ligands has a large effect on the electronic structure of the H-cluster, it seems to have no significant effect on the activity.^60^

**Table 1:** Structure and relative activities of different cofactors integrated into an [FeFe] scaffold

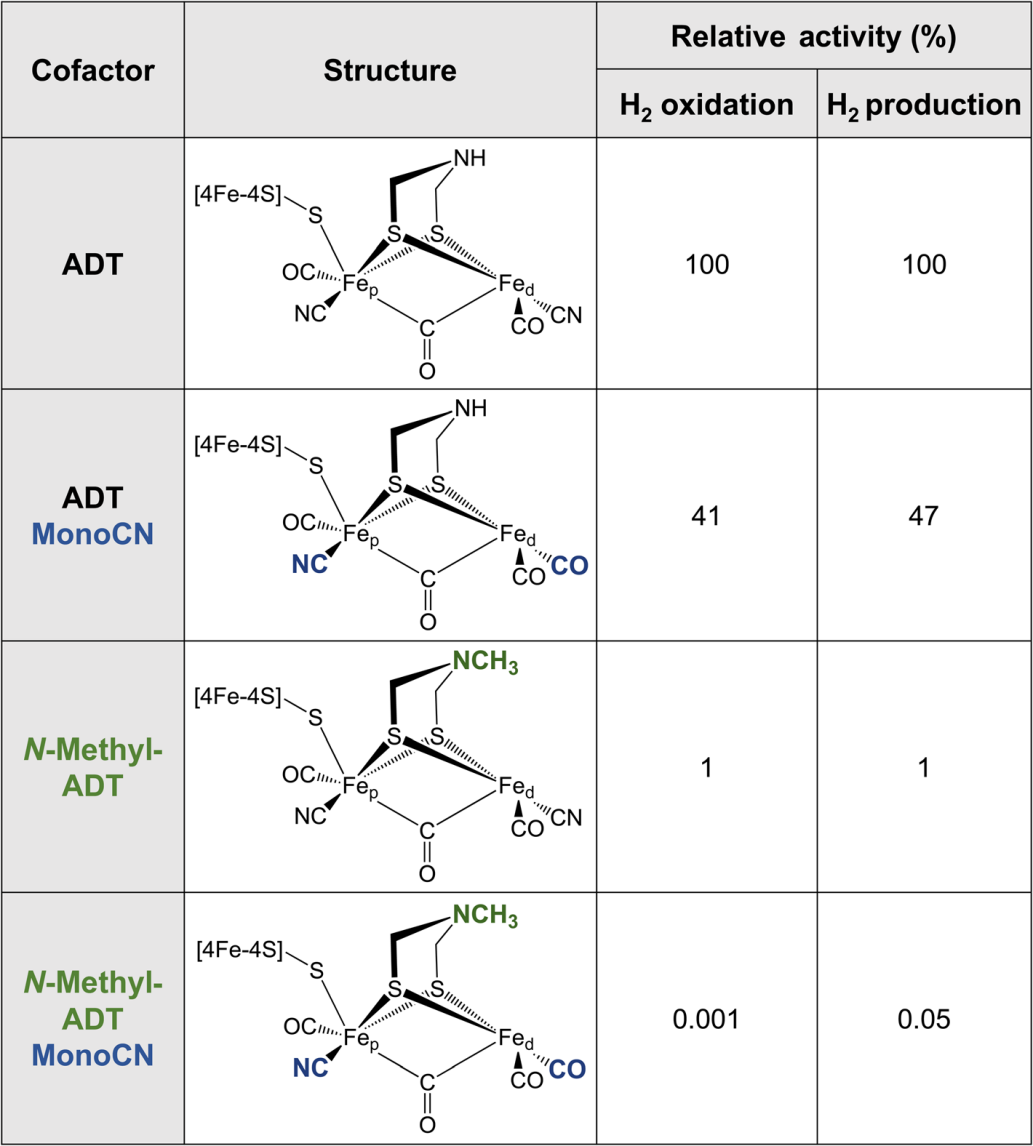

The enzyme containing an *N*-methylazadithiolate (*N*-methyl-ADT) bridge possessed 1% activity for H_2_ production and oxidation. This may indicate that the ability to form an ammonium ion, with a doubly protonated nitrogen (R-NH_2_^+^), could be crucial for activity. Interestingly, like the PDT variant, the as-isolated state of this [FeFe] hydrogenase appears to be the H_red_ state, whereas, under the same conditions, the native enzyme was in a mixture of H_ox_, H_red_, H_red_H^+^ and H_sred_H^+^ states. This indicates that the *N*-methylamine group has a lower pK_a_ and is unprotonated under these conditions (pH 8). This is counterintuitive as protonated tertiary amines are expected to be stabilised relative to protonated primary amines due to the inductive effect of the additional methyl carbon. However, the interaction with the protein matrix may play an additional role (discussed below). Another question regarding this variant is whether the methyl group points away from the open coordination site or toward it. The free complex is more stable with the methyl group pointing down toward the metals due to the anomeric effect and there will be reduced steric repulsion if the methyl group occupies space in the open coordination site. However, this would block the binding of H_2_, possibly providing another explanation for the lack of activity. Ultimately, crystal structures could reveal why this variant is so inactive.

The monocyanide form of the *N*-methyl-ADT variant was also tested and found to have 0.05% of the H_2_ production activity and 0.001% of the H_2_ oxidation activity of the native-like enzyme. This was surprising for two reasons. Firstly, *N*-methyl-ADT and monocyanide substitutions independently have little effect on the catalytic bias, but together shift the bias much more in favor of H_2_ production. Secondly, the monocyanide substitution of the ADT variant only halves the activity whereas this substitution on the *N*-methyl-ADT variant decreases the activity over 20-fold. Clearly, the factors affecting the enzyme activity are not well understood with multiple factors at play, so further investigation of cofactor variants using spectroscopic and electrochemical approaches could provide crucial insight.

**Fig. 4:**
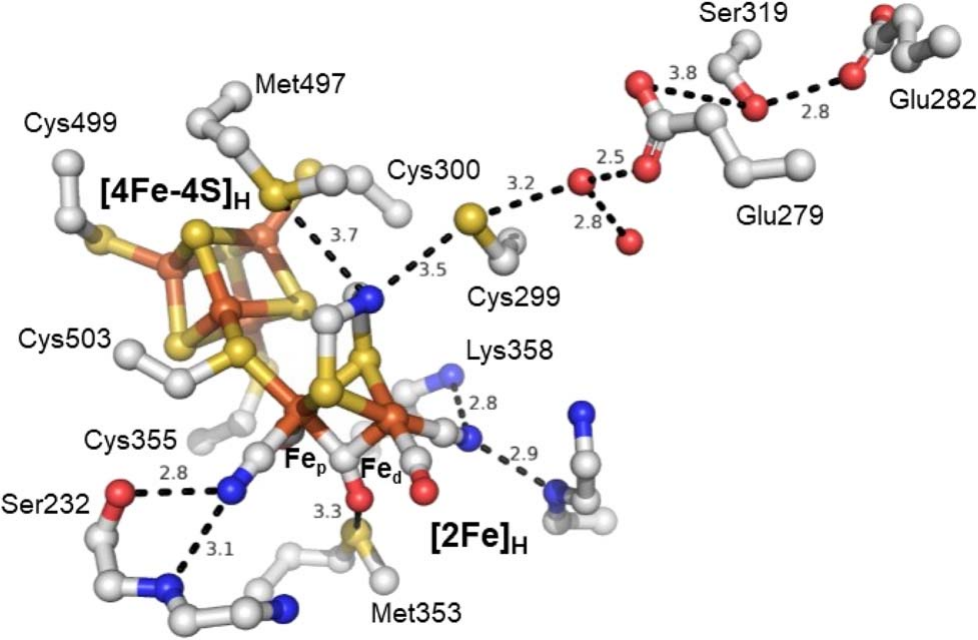
Structure of the active site H-cluster of [FeFe] hydrogenase showing the interacting amino acid residues and proton transfer pathway. Distances shown are in Å. Residue numbering is from the *C. pasteurianum* enzyme. The image was prepared using PDB ID 4XDC.^24^

The active site of an [FeFe] hydrogenase is intimately influenced by its interactions with the protein matrix. These interactions include the covalent attachment of [4Fe–4S]_H_ and [2Fe]_H_*via* a cysteine residue, hydrogen-bonding interactions to the bridging thiolate, CO, and CN^−^ ligands, and longer-range electrostatic interactions. The study by Knözer and coworkers was the first to try and understand these effects by mutating the protein matrix surrounding the H-cluster in *Cr*HydA1 and *Cp*I.^26^ They found that several surrounding amino acids play important roles in catalysis. A cysteine residue (Cys299) located within hydrogen bonding distance of the ADT ligand (Fig. 4) and thought to make up part of the proton transfer pathway was mutated to serine giving a completely inactive enzyme with unusual spectroscopic properties. A methionine (Met497), whose thioether group is positioned close to the ADT bridging ligand was mutated to leucine and gave a slightly less active enzyme. Spectroscopic analysis attributed this to a loss of the [2Fe]_H_ subcluster from the active site. Another methionine (Met353) whose thioether group is positioned close to the bridging CO ligand was mutated to leucine giving a less active enzyme. Spectroscopic analysis was consistent with a high level of intact H-cluster, indicating that the lowered activity was related to the loss of an interaction between the thioether of the methionine and the bridging CO ligand. Lastly, mutation of an arginine residue (whose guanidinium group is positioned very close to the distal CN^−^ ligand), when mutated to asparagine, gave an inactive enzyme which lacked both IR and EPR signals from the [2Fe]_H_ site. From this, it was established that some amino acids surrounding the H-cluster are catalytically relevant while others play a role in stabilising the [2Fe]_H_ subcluster.

Subsequent studies have further investigated the role of the cysteine in the proton transfer pathway, mutating it to serine,^62–65^ alanine^59,63,66^ and aspartic acid.^62,63,66,67^ The cysteine likely forms a hydrogen bond with the ADT ligand. It is generally accepted that this amino acid is responsible for directly exchanging protons between the H-cluster and the proton transfer pathway. Recent studies have also suggested that it directly influences the H-cluster's electronic structure.^59,62^ The IR spectrum of the as-isolated C169A mutant of *Cr*HydA1 is essentially identical to that of the H_ox_ state of wild-type *Cr*HydA1,^59^ but EPR spectroscopy shows that the state has one more electron than H_ox_, and is H_red_-like with IR bands shifted to higher energy. Oxidation of the C169A mutant gives an IR spectrum with higher energy IR bands and an EPR spectrum typical of H_ox_. This suggests that the C169A mutant has a more electron-deficient [2Fe]_H_ core. Similar results have been reported for the C169S mutant^65^ but interpreted differently.

A further interesting feature of the C169A and C169S mutants is that they both stabilise a terminal hydride intermediate (H_hyd_).^39,64,65^ This state is extremely persistent in these mutants and is observed during spectroelectrochemical titrations,^64^ confounding the initial kinetic proposal that a deficient proton transfer pathway slows the rate of transfer and traps the H_hyd_ state. By acting as a hydrogen bond acceptor C169 of the wild-type enzyme may increase the negative charge on the nitrogen of the ADT ligand, increasing its pKa and strengthening its basic properties. This would affect the thermodynamic hydricity at the H-cluster^68,69^ stabilising the protonated amine (H_red_H^+^ and H_sred_H^+^ states) over the protonated Fe (H_hyd_).

A third argument in support of this idea is that the C169A mutants of *Cr*HydA1, and the equivalent C178A mutant of *Dd*HydAB, can tolerate the binding of CN^−^ to the apical coordination site of the H-cluster much more readily than wild-type enzymes, whose H-clusters cannot tolerate the additional electron density and partially decompose on addition of CN^−^.^59^ The C169A and C178A mutants have no hydrogen bonding interaction with ADT, removing additional negative charge on the nitrogen. As the ADT ligand is now more electron-withdrawing the H-cluster can accept the extra electron density introduced by the additional CN^−^ ligand; the free NH groups of their ADT ligands may hydrogen bond to the exogenous CN^−^ ligand, further increasing the stability of the H-cluster.

Overall, the hypothesised hydrogen bond from the ADT ligand to the cysteine thiol helps explain several observations but is far from being conclusively demonstrated. Further experiments such as investigations of the C169A mutant containing the PDT or ODT cofactor are needed to provide additional insight.

The electron relay serves as a pathway for electrons to reach the active site but the properties of these clusters are thought to also influence catalytic activity, catalytic bias, and oxygen sensitivity. Removing the F-domain was achieved for the *Clostridium acetobutylicum*^70^ and *Megasphaera elsdenii*^71^ [FeFe] hydrogenases, leading to loss of activity. For the *M. elsdenii* enzyme, the catalytic bias was also affected. In the *D. desulfuricans* [FeFe] hydrogenase, it was observed that reduction of the F-clusters influenced the redox state of the H-cluster through electrostatic repulsion.^34^ This effect destabilised states in which the [4Fe–4S]_H_ subcluster was reduced such as H_red_ and H_sred_H^+^. These states are still likely to form transiently during catalysis even if they are not observed during thermodynamic titrations.

Thus, it has been made clear that the H-cluster cannot be considered an isolated entity as it is intimately connected to the protein scaffold. This has an enormous influence on the electronic and geometric structure through various types of interactions. These interactions are only now beginning to be understood for a select group of model enzymes. It is very likely that studying the diversity of [FeFe] hydrogenases in nature will reveal additional insight and possibly specific evolutionary adaptations that modify the H-cluster for specific functions.

## The diversity of [FeFe] hydrogenases: prototypical, ancestral and sensory type

**Fig. 5:**
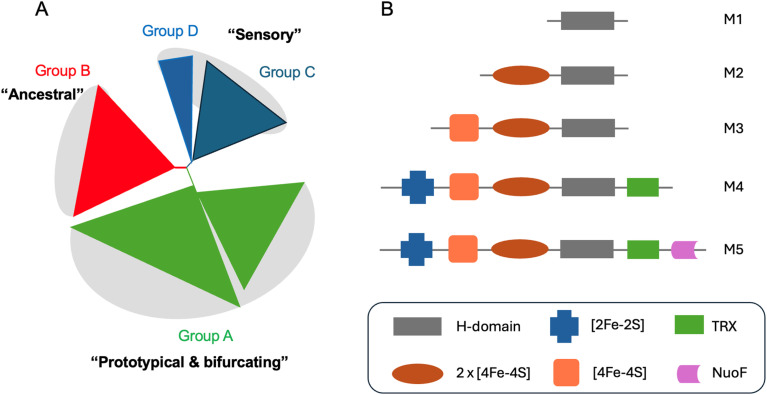
(A) Simplified view of the [FeFe] hydrogenases phylogeny diagram proposed by Greening *et al.* (2016).^74^ (B) Biodiversity of [FeFe] hydrogenases represented by five main subclasses named as M1–5. M stands for monomeric and number indicates the size. Legend: TRX – thioredoxin, NuoF – NADH: ubiquinone oxidoreductase chain F.

In recent years it has become clear that there is huge diversity of [FeFe] hydrogenases in nature and so far, only a tiny fraction of this diversity has been studied. In 2001, Vignais, Billoud and Jacques reviewed the classification and phylogeny of hydrogenases based on the available data.^72^ They presented examples of [FeFe] hydrogenases containing only the catalytic H-domain (*e.g.* from the green algae *C. reinhardtii* and *Chlorella fusca*), containing accessory iron–sulfur cluster domains (*e.g.* from *M. elsdenii* and *C. pasteurianum*), possessing additional subunits (*e.g.* from *Desulfovibrio fructosovorans* and *Thermotoga maritima*) and even an enzyme with a similar composition to the *D. fructosovorans* and *T. maritima* enzymes but where the enzyme's subunits are fused into one polypeptide. Then, in 2007, Meyer classified [FeFe] hydrogenases into the more familiar M1–M5 subgroups, where M stands for monomeric.^73^ M1 indicates the presence of the H-domain only and M2–M5 indicate the presence of additional iron–sulfur cluster domains (Fig. 5). In this article, Meyer performed extensive phylogenetic analysis with a subset of the [FeFe] hydrogenase sequences available and found that the majority of sequences cluster together and include sequences from the M1–M5 groups.

Later, Calusinska *et al.* performed a phylogenetic analysis of hydrogenases with a focus on *clostridia* and found that [FeFe] hydrogenases could be divided into several groups (A, B, C and D), of which the first two groups were further subdivided (A1–8 and B1–3).^75^ Each of these groups corresponded well to phylogenetic groups identified previously by Meyer. Group C was remarked as consisting of dimeric PAS/PAC sensory domain containing hydrogenases and group D as consisting of putative hydrogenases. In 2016, Greening *et al.* carried out an even more extensive phylogenetic analysis on 1223 [FeFe] hydrogenase sequences and classified them similarly to Calusinka where group A was subdivided into four groups and group D was integrated into group C (Fig. 5).^74^ Overall, these studies show an enormous diversity of [FeFe] hydrogenase sequences of which very few have been investigated.

Group A, also known as the prototypical group, includes the vast majority of [FeFe] hydrogenases studied to date, and the only group for which structures are available. It includes *Cp*I^14,76^ from *C. pasteurianum*, *Dd*HydAB^15,47^ from *D. desulfuricans* and *Cr*HydA1 ^77^ from *C. reinhardtii*. Also included is the class of electron-bifurcating [FeFe] hydrogenases^78,79^ that reversibly reduce protons using NADH and ferredoxin synergistically, as well as a class of hydrogen uptake [FeFe] hydrogenases, A3, which includes *Cp*II^80,81^ from *C. pasteurianum*, and the uniquely oxygen-stable A5 [FeFe] hydrogenase, *Cb*A5H, from *Clostridium beijerinckii*.^82^

Group B, also known as the ancestral group, is much more poorly studied, with only a few characterised examples including the functionally and spectroscopically characterised *Cp*III from *C. pasteurianum* and the less well-characterised HydA2 from *Clostridium acetobutylicum*.

Group C, also known as the sensory group, is also poorly studied but two examples, HydS from *Thermotoga maritima* (*Tm*HydS)^44,83^ and HydS from *Thermoanaerobacter mathranii* (*Tam*HydS)^84–86^ have recently been produced and characterised both functionally and spectroscopically. These enzyme have very low activity and, at least in the case of *Tam*HydS, show extremely irreversible electrochemical behavior.^84^ They are theorised to have a sensing function, presumably hydrogen, but, as yet, the mechanism of signal transduction is unknown.

To provide one example of how different [FeFe] hydrogenases from different groups can be, *Tm*HydS and *Tam*HydS both lack the crucial amino acids of the proton-transfer pathway identified in group A [FeFe] hydrogenases including a cysteine that directly interacts with the ADT ligand and is thought to be the direct proton donor/acceptor of the H-cluster. In the case of *Tam*HydS an alternative proton transfer pathway has been identified.^87^ A proton transfer pathway is yet to be identified for *Tm*HydS despite intense investigation.^83^

There is still much to learn about the wider diversity of [FeFe] hydrogenases. A crucial part of future research will be to characterise enzymes from groups B, C and the more diverse branches of group A. Understanding their unique physiological functions and how these influence their protein sequences and thus the structure and properties of the active site and accessory clusters is crucial to understanding how enzyme efficiency is maximised. By understanding these structure–function relationships we will learn more about designing optimal H_2_ oxidation and H_2_ production catalysts based on iron.

## Future outlook: the missing pieces of the catalytic cycle

### The hydride state, H_hyd_

As discussed above, the first steps of the catalytic cycle in the H_2_ production direction, namely reduction, protonation and further reduction have been comprehensively studied, and we are reaching a point where these stages of the catalytic cycle are understood. It has been proposed that another form of the two-electron reduced, protonated state exists, called H_hyd_, in which there is a terminal hydride bound to the apical position on Fe_d_. How exactly this state forms is not clear but the following observations have been made:

(1) H_hyd_ can be formed at low pH in the presence of high concentrations of sodium dithionite (Na_2_S_2_O_4_).^39^ This was taken as evidence that this state is transiently formed under conditions of high turnover.

(2) H_hyd_ can be formed under equilibrium conditions in enzymes where there have been mutations made to the terminal cysteine in the proton-transfer pathway^65^ or where the ADT bridging ligand is substituted with oxadithiolate (ODT).^39^ It has been assumed that these modifications restrict proton transfer and so the terminal hydride that is formed as an intermediate cannot be protonated to make H_2_.

(3) Alternative forms of H_hyd_ named H_hyd:ox_ and H_hyd:red_ can be formed upon illumination of H_red_H^+^ and H_sred_H^+^, respectively, under cryogenic conditions.^43^ These results may indicate that the H_hyd_ states are less stable, tautomeric forms of H_red_H^+^ and H_sred_H^+^, and so energy must be supplied to enable proton transfer from the nitrogen base to Fe_d_.

(4) Electrochemical titrations of *Cp*I crystals have shown to populate hydride species.^88^

Another explanation for the formation of H_hyd_ in the disrupted proton-transfer mutants and ODT enzyme stands. Both have less basic bridgeheads; mutation of the cysteine may influence the basicity of the ADT amine through disruption of hydrogen bonding and the ether bridgehead in ODT is clearly less basic than the native amine. A follow-up question then is why the PDT enzyme, in which the amine is substituted with methylene, does not behave in the same way as the ODT enzyme. This may be explained by differences in the redox potential of the diiron clusters between PDT and ODT, where ODT ought to be more positive than PDT. One might then expect that PDT can indeed form H_hyd_ but at more negative potentials than ODT, so far not accessed experimentally.

But what is the difference between H_hyd:ox_ and H_hyd:red_ and the H_hyd_ states observed at low pH and in mutants? Table 2 compares the IR bands and EPR *g*-values of the H_hyd_ states so far identified in *Cr*HydA1.

**Table 2:** Spectroscopic signatures of H_hyd_ states reported in the literature for the HydA1 [FeFe] hydrogenase from *Chlamydomonas reinhardtii*

Name	IR							EPR			Ref.
	CN_p_	CN_d_	CO_p_	CO_d_	CO_b_	Average	Difference to H_hyd_	*g*_1_	*g*_2_	*g*_3_	
H_hyd_	2082^a^ (2088)	2068^a^ (2075)	1978 (1979)	1960 (1961)	1860 (1861)	1990 (1993)	—	2.077 (2.080)	1.935 (1.941)	1.880 (1.884)	39 (89)
							+3				
H_hyd:ox_	2092	2086	1983	1954	1865	1996	+6 (+3)	n.a.	n.a.	n.a.	43
H_hyd:red_	2087	2078	1972	1954	1851	1988	−2 (−5)	2.069	1.938	1.880	43
H_hyd(ODT)_	2081^a^ (2090)	2076 (2075)	1980 (1980)	1962 (1963)	1868 (1869)	1993 (1995)	+3 (0)	(2.069)	(1.941)	(1.879)	39 (40)
							+5 (+2)				
H_hyd(C169A)_	2082^a^ (2089)	2068^a^ (2076)	1978 (1980)	1962 (1962)	1862 (1864)	1990	0 (−3)	(2.075)	(1.942)	(1.884)	39 (59)
						1994	+4 (+1)				
H_hyd(C169S)_	n.r.	n.r.	1977	1960	1860			2.068	1.943	1.881	65
H_hyd(E279A)_	2082	2068	1984	1970	1858	1992	+2 (−1)	n.r.	n.r.	n.r.	39
H_hydH+(C169S)_	n.r.	n.r.	1987	1967	1874			2.065^b^	1.969^b^	1.906^b^	64

a Values reported in ref. 39 deviate substantially (>6 cm^−1^) from other ref. 40, 59, 66 and 89.

b The same EPR spectrum has been assigned to a “H_trans_-like” state in ref. 90 and a similar EPR spectrum was reported for the CN^−^-bound “H_trans_-like” state in the C169A mutant of *Cr*HydA1 (*g* = 2.068, 1.977, 1.916) in ref. 59.

The first thing to note is some discrepancies between the IR band frequencies measured for what should be identical H_hyd_ states in different studies. In most cases these discrepancies are small (>3 cm^−1^) but for the CN^−^ bands they appear to be larger. In general H_hyd:red_ appears to have the most red-shifted IR bands indicating the most electron-rich [2Fe]_H_ site. Meanwhile, H_hyd:ox_ has more blue-shifted IR bands. The differences between these two states are thought to be due to the difference in the redox state of [4Fe–4S]_H_, which is oxidised in H_hyd:ox_ and reduced in H_hyd:red_. The H_hyd_ state formed at low pH, on the other hand, possesses IR bands that are blue-shifted relative to H_hyd:red_ but red-shifted relative to H_hyd:ox_, yet is also proposed to have a reduced [4Fe–4S]_H_ like H_hyd:red_. Clearly, there is something about the structure around the H-cluster in H_hyd_ that is different. Stripp and coworkers proposed that H_hyd_ has a protonated [4Fe–4S]_H_,^36,66^ which would shift some of the electron density from [2Fe]_H_ back onto [4Fe–4S]_H_. This idea agrees with the observation of this state at low pH,^39^ although the protonation of the [4Fe–4S]_H_ cluster in other catalytic states is still disputed.^37,38^ The H_hyd_ states formed in the ODT enzyme and a C169A mutant have similar IR bands to the H_hyd_ state formed at low pH.^39^ Again, these could represent states with protonated [4Fe–4S]_H_ clusters, however, these H_hyd_ states are stable at neutral pH. Since neither of these changes (substitution of ADT with ODT or mutation of Cys169 to Ala) should influence the pK_a_ of [4Fe–4S]_H_ it seems unlikely that these states would represent protonated [4Fe–4S]_H_ states. Instead, an alternative explanation could be that the removal of the hydrogen-bonding interaction between the ADT amine and the cysteine thiol group influences the electronic structure of the H-cluster in such a way that disfavours protonation of the bridging ADT and favours terminal hydride formation on Fe_d_. If this is the case, then could this also explain the stabilisation of H_hyd_ at low pH and the differences in the IR signatures of H_hyd_*vs.* H_hyd:red_? Evidence in this direction comes from comparison of the H_ox_ and H_red_ states in the ODT enzyme and the C169A mutant (Table 3).

**Table 3:** IR spectroscopic signatures of H_ox_ and H_red_ in *Cr*HydA1 WT ADT, ODT and C169A ADT

Name	IR							Ref.
	CN_p_	CN_d_	CO_p_	CO_d_	CO_b_	Average	Difference to H_ox_	
H_ox_ (WT/ADT)	2088	2072	1964	1940	1804	1974	—	91
H_ox_ (WT/ODT)	2092	2076	1970	1947	1811	1979	+5	91
H_ox_ (C169A/ADT)	2092^a^ (n.r.)	2075^a^ (n.r.)	1972^a^ (1972)	1946^a^ (1948)	1813^a^ (1815)	1980	+6	66 (59)
H_red_ (WT/ADT)	2084	2066	1962	1933	1792	1967	−7	91
H_red_ (WT/ODT)	2083	2070	1964	1943	1804	1973	−1	91
H_red_ (C169A/ADT)	2089^b^	2068^b^	1971^b^	1939^b^	1804^b^	1974	0	59

a This spectrum was actually assigned to H_ox_H in ref. 66. However, in ref. 59 it was observed that this state formed under conditions typical for forming H_ox_ and was associated with an H_ox_-like EPR spectrum.

b This state was assigned to H_red_ in ref. 66. However, in ref. 59 it was observed that this state formed under conditions typical for forming H_red_ and was found to be an EPR-silent state, untypical for H_ox_

From Table 3, the H_ox_ spectra for ODT and C169A are blue-shifted compared to H_ox_ in WT ADT, while the H_red_ spectra for ODT and C169A are like H_ox_ in WT ADT but blue-shifted compared to H_red_ in WT ADT. This indicates that IR spectra for the ODT and C169A enzymes are blue-shifted compared to their WT counterparts.

### Have we observed H_hyd_H^+^ and/or H_ox_(H_2_)?

While there have been some reports of H_hyd_H^+^ in the literature, these are yet to be substantiated with direct evidence and mostly rely on observed IR and EPR spectra that are somewhat similar to H_hyd_ but blue-shifted (in the case of the IR spectra). In 2014, Mulder observed IR bands at 1987, 1967 and 1874 cm^−1^ in the C169S mutant of *Cr*HydA1, where the bridging CO shifted to 1964 cm^−1^ in D_2_O and was associated with an EPR spectrum with *g*-values of 2.065, 1.969 and 1.906.^65^ DFT calculations supported an assignment to H_hyd_H^+^, with a protonated ADT and a reduced [4Fe–4S]_H_ cluster. In 2021, Mészáros observed a similar IR spectrum with bands at 1988, 1959 and 1975 cm^−1^ for WT *Cr*HydA1, associated with an EPR spectrum with *g*-values of 2.073, 1.935 and 1.881 and assigned this to the same H_hyd_H^+^ state.^92^ While there are some similarities between the spectroscopic properties of these two putative H_hyd_H^+^ states, they are clearly very different to the H_hyd_ states of WT and C169S *Cr*HydA1, whose IR and EPR spectra are nearly identical. Thus, the assignment of these two new signals to H_hyd_H^+^ seems premature. Furthermore, it would be surprising if the H-cluster of H_hyd_H^+^ retained the same overall electronic structure as that of H_hyd_. One might expect the protonation of the ADT ligand to trigger electron transfer yielding a [4Fe–4S]^2+^-[Fe(i)Fe(ii)H^−^] electronic structure in which the reducing equivalent is on [2Fe]_H_. Such a state would be primed for H_2_ formation yielding the H_ox_ state.

While H_hyd_H^+^ stabilisation in mutants may be achievable, it seems unlikely that it can be stabilised in WT enzymes due to their exceptionally fast turnover rates. The same is probable for any potential H_ox_(H_2_) intermediate. A possible way of capturing these states would be to use ultra-fast spectroscopic techniques such as pump-probe IR, which has been coupled to some kind of method for rapidly triggering catalysis. The Dyer group have pioneered methods for using CdS/CdSe dot-in-rod photosensitiser systems coupled to redox mediators for light-triggered reduction of [FeFe] hydrogenases among other enzymes.^48,52^ Work with *Cr*HydA1 showed that the enzyme converted from H_ox_ to H_red_ on a timescale of 10 to 100 μs; over the same time-period, H_red_H^+^ and H_sred_H^+^ were also formed. H_hyd_ was formed between 50 and 500 μs. All states eventually decayed reforming H_ox_ on timescales similar to those expected based on the known catalytic rates for H_2_ production by *Cr*HydA1 (≈1000 s^−1^ or 1 μs^−1^). A caveat of this study was that the starting state of the enzyme was a mixture of H_ox_, H_red_ and H_red_H^+^, which complicated the kinetic analysis as single electron reduction will convert H_ox_ to H_red_/H_red_H^+^ and reduce H_red_/H_red_H^+^ to H_sred_H^+^/H_hyd_, and so the kinetics of H_red_/H_red_H^+^ formation are a convolution of both a formation and decay rate. Furthermore, in a subsequent study it was found that increasing the protein concentration led to faster kinetics indicating a diffusion limitation.^51^ Accordingly, this method could be enhanced by engineering photosensitisers directly onto [FeFe] hydrogenases, as has been previously shown.^55–57,93,94^

### Have we already observed an H_2_-bound, H_ox_(H_2_), intermediate?

As we are unsure of what we are looking for, that is entirely possible. What do we expect it to look like? Here, some clues can be acquired from investigation of synthetic η^2^-H_2_ metal complexes.^95^ Many η^2^-H_2_ mono-metallic complexes have been studied over the years but examples of bimetallic complexes like [2Fe]_H_ that stably bind η^2^-H_2_ are rarer.^96–101^ Generally, dihydrogen complexes are identified through structural characterization (X-ray or neutron crystallography), vibrational spectroscopy (IR, Raman, inelastic neutron scattering) and NMR spectroscopy. Free H_2_ in the gas phase has a vibrational frequency of around 4100 cm^−1^ (ref. 102) and the transition is IR invisible due to the absence of a change in dipole moment. Binding of H_2_ to metals gives IR active modes due to the dipolar character of the metal–hydrogen bonds and the coupling of the H–H stretch to other vibrations. A range of complexes show vibrational modes in the 2200–3200, 1100–1700 and 400–1000 cm^−1^ regions.^95^ Due to the low intensity of these peaks and their potential overlap with other bands from the protein matrix, the possibility of observing a dihydrogen bound species by IR is low. In NMR, metal-bound dihydrogen presents with chemical shifts in the 2.5 to −31 ppm region, which overlaps with the chemical shifts of metal-hydrides.^95^ For reference, the [FeFe] hydrogenase H_hyd_ state gives a peak at −9.6 ppm.^103^ However, observation of *J*_HD_ coupling or short *T*_1_ relaxation times can provide support for a dihydrogen species. However, in reality, this can only be achieved with *S* = 0 systems as unpaired electrons tend to produce NMR line-broadening, as would be expected for a putative H_ox_(H_2_) intermediate.

Specifically, for [FeFe] hydrogenases, EPR spectroscopy may provide a valuable tool for identifying and characterizing H_2_-aducts. The Peters and Hoffmann groups measured EPR and ENDOR on an H_2_-bound Fe(i) complex, which revealed a single electron–nuclear hyperfine interaction with a coupling tensor of [2.3, −40.6, −37.8] with both isotropic and anisotropic components.^104^

Returning to IR, it might be possible to observe the H_2_-bound intermediate indirectly through changes to the CO and CN^−^ ligand vibrations upon H_2_ binding. Side-on binding of H_2_ has both a σ-donation and π-backdonation contribution. Binding of hydrogen to monometallic carbonyl complexes causes a small blue shift in the vibrational frequency of the carbonyl IR bands, likely reflecting the decreased backdonation due to some of this electron density being donated into the H_2_ σ*.^95^ A similar effect is seen in CO binding to the [FeFe] hydrogenase H-cluster forming the H_ox_–CO state; the IR band of the bridging CO is blue-shifted by around 10 cm^−1^.^13^ So, it can be postulated that an H_ox_(H_2_) state may show similar blue shifted IR bands.

NRVS has been used extensively to probe the Fe–H vibrations of the H_hyd_ states but could also be a tool to explore potential H_ox_(H_2_) states. For an Fe-η^2^-H_2_-hydride complex an intense NRVS symmetric Fe–H_2_ stretching mode was observed at 1052 cm^−1^ that shifts to 781 cm^−1^ upon D_2_ substitution.^105^ In addition, the weaker antisymmetric stretch mode was observed at 1774 cm^−1^ and bending modes were observed at 558, 584, 733 and 823 cm^−1^. These results suggest that in principle, an H_2_ bound intermediate of an [FeFe] hydrogenase could be probed with NRVS provided it is sufficiently enriched.

Lastly, X-ray crystallography is generally not considered as an effective method for observing protons due to their low electron density and bond libration effects that give anomalous bond lengths. Despite this, it has been used to provide evidence for the location of hydrides and protons in [NiFe] hydrogenase enzymes due to sub-angstrom resolution quality of the data.^106^ Alternatively, neutron diffraction could be employed, as neutrons are scattered predominantly by the nucleus and the coherent neutron scattering length of hydrogen is similar to heavy atoms. However, due to the negative sign of the scattering length of ^1^H and the large amount of incoherent scattering it is much easier to obtain information on the location of deuterium. Regardless, due to the weak intensity of neutron sources very large crystals are needed and data collection times are extremely long. Recently, a neutron structure was published for an oxidised form of [NiFe] hydrogenase,^107^ however, this technique is yet to reveal the locations of hydrides and protons from hydrogen splitting. The size of the crystals needed (at least 1 mm^3^) on top of the requirement for deuteration continues to cause problems for neutron diffraction experiments. Nevertheless, it is likely that the next few years will see the publication of neutron and subangstrom resolution X-ray structures of [FeFe] hydrogenases, revealing the locations of protons. If these can be carried out on the H_hyd_ states or potential H_hyd_H^+^ and H_ox_(H_2_) intermediates, these structures will provide unprecedented insight into our understanding of biological H_2_ activation.

### New tools: CryoEM and its potential in hydrogenase research

Electron imaging visualises electrostatic potential, which depends on the positions of both nuclei and electrons.^108^ Thus, hydrogens can be observed more clearly in cryoEM than in X-ray crystallography. Another advantage of cryoEM is that samples do not need to be crystallised. However, there is currently a size limit of around 50 kDa, below which obtaining cryoEM structures is exceptionally difficult, but still possible. In recent years, several cryoEM structures of hydrogenases have been published including various electron-bifucating [FeFe] hydrogenases.^109–113^ One structure of a [NiFe] hydrogenase reached 1.52 Å resolution, potentially paving the way toward resolving hydrogen atoms.^113^

### A multiple catalytic pathways scenario?

**Fig. 6:**
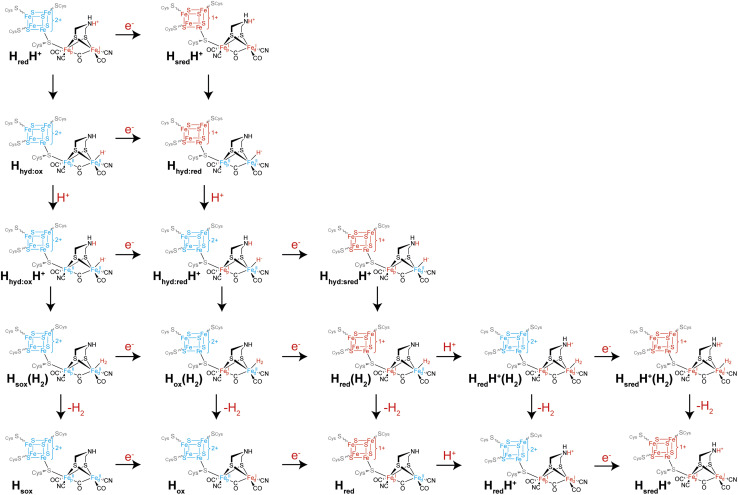
Possible alternative pathways taken by the H-cluster from the H_red_H^+^ state (top left), which can proceed by reduction to the H_sred_H^+^ state (horizontal arrow) or tautomerism to the H_hyd:ox_ state (vertical arrow). The H_sred_H^+^ state can also tautomerise (vertical arrow), yielding the H_hyd:red_ state. Both forms of the H_hyd_ state can then be protonated (vertical arrows) and the two possible forms of H_hyd_H^+^ can then undergo reduction (horizontal arrows) or tautomerism (vertical arrows), and so on.

Another intriguing idea is that of multiple catalytic pathways. Earlier in this article, two hypotheses for the catalytic cycle of [FeFe] hydrogenase were presented, both with some evidence in support of them. However, it is possible that, to a certain extent, elements of both cycles are true, and that alternative pathways are taken by [FeFe] hydrogenases depending on (i) the conditions and (ii) which enzyme is being studied. It has already been demonstrated for *Cr*HydA1 that at least three different forms of the H_hyd_ state exist including the ‘classical’ H_hyd_ observed at low pH and H_hyd:ox_ and H_hyd:red_ observed at high pH, mainly under cryogenic conditions and illumination but also thought to contribute at room temperature. During proton reduction, from the H_red_H^+^ state there are multiple potential pathways that can be taken (Fig. 6): if electron transfer is slow then tautomerization to H_hyd:ox_ may be faster than reduction to H_sred_H^+^. H_hyd:ox_ could then be reduced to H_hyd:red_ or at low pH become protonated to a putative H_hyd:ox_H^+^ state, which could either become further reduced to a putative H_hyd:red_H^+^ state or, again if electron transfer is slow, directly form H_2_. Formation of H_2_ from a putative H_hyd:ox_H^+^ state would leave the [4Fe–4S]_H_ cluster oxidised and the [2Fe]_H_ cluster in an overoxidised Fe(ii)Fe(ii) oxidation state, a putative “H_sox_^”^ state, which may not be stable. Thus, it can already be seen that depending on the electron supply, pH, and enzyme properties such as redox potentials and pK_a_ values, several pathways from H_red_H^+^ may exist. Since H_sred_H^+^ cannot be further reduced or protonated, it can only tautomerise to H_hyd:red_. H_hyd:red_ is unlikely to be reducible but will become protonated to give H_hyd:red_H^+^. H_hyd:red_H^+^ formation is likely to involve PCET where [4Fe–4S]_H_ transfers an electron to [2Fe]_H_. In principle, H_hyd:red_H^+^ could be further reduced to give H_hyd:sred_H^+^ where both the [4Fe–4S]_H_ and [2Fe]_H_ clusters are reduced, which would in turn form H_2_ bound to the H_red_ state in a H_red_(H_2_) state. Likewise, it would also be possible to reduce the H_ox_(H_2_) state before releasing H_2_ giving H_red_(H_2_). Such states could potentially be observed under conditions where there is a very fast electron supply. One could also envisage additional states such as the protonated H_ox_ state (H_ox_H^+^) or the deprotonated H_sred_H^+^ (H_sred_) state that could follow additional pathways. Whether any of these states are populated during turnover, even under extreme conditions, is not known but it is speculated to be the case based on electrochemical studies of amino acid variants.^46^ It may also be possible that accessing some of these alternative pathways is responsible for phenomena observed under extreme conditions during electrochemistry such as high potential inactivation^114^ and low potential inactivation.^45^

It could also be possible that different pathways are followed depending on the direction of catalysis. For example, if interconversion of the H_red_H^+^ and H_sred_H^+^ states with their H_hyd_ tautomers is slow compared with electron transfer, then during proton reduction the pathway H_red_H^+^ → H_sred_H^+^ → H_hyd:red_ would be followed while the H_hyd:red_ → H_hyd:ox_ → H_red_H^+^ pathway would be followed during H_2_ oxidation. Determining whether [FeFe] hydrogenases do indeed follow multiple pathways depending on conditions requires the ability to carefully control the rate of electron supply while monitoring the composition of states using spectroscopy. This is very difficult using standard spectroscopic approaches where electrons are exchanged with the enzyme using mediators. Instead, an approach is needed where the enzyme is directly attached to an electrode surface. The techniques of surface enhanced IR spectroscopy (SEIRAS) and protein film infrared electrochemistry (PFIRE) are designed to do this.^115^ While [NiFe] hydrogenases have been quite extensively studied with SEIRAS^116–119^ and also by PFIRE,^120,121^ there are only two examples of SEIRAS being employed with an [FeFe] hydrogenase, specifically *Cr*HydA1,^122,123^ and no examples of PFIRE applied to [FeFe] hydrogenases. In both SEIRAS studies only the spectra of the protein backbone amides could be observed and there was no evidence of the active site CO and CN^−^ ligands, even though catalytic activity was observed. Thus, there is potential for this technique and for PFIRE to be incredibly useful in future studies, to particularly allow *operando* conditions.

## Conclusion

[FeFe] hydrogenases are incredibly fascinating yet complex enzymes capable of extremely high turnover frequencies with exceptional efficiency and reversibility. How this is achieved is still not completely understood despite decades of research. In this perspective we have provided a summary of where we are now in terms of our understanding, how we have gotten here and where we think the field is moving and how it is going to get there, particularly highlighting the main challenges. The catalytic cycle of [FeFe] hydrogenases has been a difficult puzzle to build, but we believe that enough pieces are in place to provide a good overview of the picture we are putting together. However, the next pieces of the puzzle will be the most challenging to assemble, requiring new techniques and approaches or combinations of existing techniques. The promise of being able to design new catalytic materials motivates scientists to keep working on this challenging puzzle and we expect the next few years will see substantial progress toward this goal.

## Author contributions

All authors contributed to the conceptualisation, literature search, writing and figure making of the perspective and were involved in revising, editing, and proofreading the initial and revised versions.

## Conflicts of interest

There are no conflicts to declare.
